# Diffusional Kurtosis Imaging in the Diffusion Imaging in Python Project

**DOI:** 10.3389/fnhum.2021.675433

**Published:** 2021-07-19

**Authors:** Rafael Neto Henriques, Marta M. Correia, Maurizio Marrale, Elizabeth Huber, John Kruper, Serge Koudoro, Jason D. Yeatman, Eleftherios Garyfallidis, Ariel Rokem

**Affiliations:** ^1^Champalimaud Research, Champalimaud Centre for the Unknown, Lisbon, Portugal; ^2^Cognition and Brain Sciences Unit, University of Cambridge, Cambridge, United Kingdom; ^3^Department of Physics and Chemistry “Emilio Segrè”, University of Palermo, Palermo, Italy; ^4^National Institute for Nuclear Physics (INFN), Catania Division, Catania, Italy; ^5^Department of Speech and Hearing, Institute for Learning and Brain Science, University of Washington, Seattle, WA, United States; ^6^Department of Psychology and eScience Institute, The University of Washington, Seattle, WA, United States; ^7^Department of Intelligent Systems Engineering, Luddy School of Informatics, Computer Science and Engineering, Indiana University, Bloomington, IN, United States; ^8^Department of Pediatrics, Graduate School of Education, Stanford University, Stanford, CA, United States

**Keywords:** MRI, diffusion MRI, DKI, DTI, microstructure, open-source software, biophysics, python

## Abstract

Diffusion-weighted magnetic resonance imaging (dMRI) measurements and models provide information about brain connectivity and are sensitive to the physical properties of tissue microstructure. Diffusional Kurtosis Imaging (DKI) quantifies the degree of non-Gaussian diffusion in biological tissue from dMRI. These estimates are of interest because they were shown to be more sensitive to microstructural alterations in health and diseases than measures based on the total anisotropy of diffusion which are highly confounded by tissue dispersion and fiber crossings. In this work, we implemented DKI in the Diffusion in Python (DIPY) project—a large collaborative open-source project which aims to provide well-tested, well-documented and comprehensive implementation of different dMRI techniques. We demonstrate the functionality of our methods in numerical simulations with known ground truth parameters and in openly available datasets. A particular strength of our DKI implementations is that it pursues several extensions of the model that connect it explicitly with microstructural models and the reconstruction of 3D white matter fiber bundles (tractography). For instance, our implementations include DKI-based microstructural models that allow the estimation of biophysical parameters, such as axonal water fraction. Moreover, we illustrate how DKI provides more general characterization of non-Gaussian diffusion compatible with complex white matter fiber architectures and gray matter, and we include a novel mean kurtosis index that is invariant to the confounding effects due to tissue dispersion. In summary, DKI in DIPY provides a well-tested, well-documented and comprehensive reference implementation for DKI. It provides a platform for wider use of DKI in research on brain disorders and in cognitive neuroscience.

## 1. Introduction

Diffusion-weighted magnetic resonance imaging (dMRI) uses a pair of directional gradient pulses to induce rephasing of proton spins, which depends on the motion of water molecules within each measurement voxel (Stejskal and Tanner, 1965; Le Bihan and Breton, 1985). Although dMRI measurements are typically made within voxels on the order of millimeters in size, they provide a view into the microstructural properties of human tissue *in vivo*. This is because the image contrast provided by dMRI is sensitive to micron-scale distances that are probed through the random motion of water within a small amount of time between the two gradient pulses (Kiselev, 2021).

The dMRI signal in each voxel is typically approximated as a three-dimensional Gaussian distribution (Basser et al., 1994; Le Bihan and Johansen-Berg, 2012), by estimating a 2nd order tensor in every voxel. In addition to the directional information about the principal diffusion direction of the Gaussian distribution, which can be used for tractography (Mori et al., 1999; Jones, 2008), this 2nd order tensor can be used to extract scalar measures, such as the mean diffusivity (MD) and the diffusion fractional anisotropy (FA) (Basser and Pierpaoli, 1996). The diffusion tensor imaging (DTI) model provides both an accurate fit to the dMRI signal in a wide range of experimental conditions (Rokem et al., 2015), as well as useful information to probe tissue maturation or degeneration (e.g., Pfefferbaum et al., 2000; Moseley, 2002; Lebel and Beaulieu, 2011; Le Bihan and Johansen-Berg, 2012). However, in many important cases, it is known to be systematically biased (Jones et al., 2013; De Santis et al., 2014; Henriques et al., 2015) and it does not fully represent the diffusion properties of multiple different populations of water molecules inside complex biological tissue (Frank, 2001) rendering it ensitive to confounding factors such as the orientation dispersion of tissue components (Wheeler-Kingshott and Cercignani, 2009; Henriques et al., 2015), as well as the parameters of the MRI acquisition (Jones and Basser, 2004).

As an attempt to overcome these limitations of DTI, several mechanistic models directly relate diffusion properties with specific microstructural features (e.g., Assaf and Basser, 2005; Jespersen et al., 2007; Fieremans et al., 2011; Nilsson et al., 2012; Zhang et al., 2012), but improper assumptions can compromise the validity of these models (Lampinen et al., 2017, 2019; Henriques et al., 2019). To avoid misleading interpretation, a complete characterization of water diffusion in biological tissues can be obtained using phenomenological models, which are also known as signal representation techniques (Novikov et al., 2018a). Diffusional kurtosis imaging (DKI) is a phenomenological model that directly estimates the degree to which water diffusion deviates from a single Gaussian component (Jensen et al., 2005). Describing water diffusion in every voxel as an infinite mixture of Gaussian components, rather than a single Gaussian, the excess-kurtosis measured by DKI can be directly related to the variance of apparent diffusivities across different tissue components (Jensen et al., 2005; Jensen and Helpern, 2010; Fieremans et al., 2011). DKI is also sensitive to non-Gaussian diffusion effects due to the interaction of water molecules with boundaries (e.g., cell membranes or myelin sheaths) and obstacles (e.g., organelles, macromolecules) (Callaghan et al., 1991; Paulsen et al., 2015; Dhital et al., 2018; Jespersen, 2018; Henriques et al., 2020a). This means that the scalars provided by DKI closely relate to microstructural alterations in health and in brain diseases (Grossman et al., 2012; Hui et al., 2012; Fieremans et al., 2013; Benitez et al., 2014; Rudrapatna et al., 2014; Steven et al., 2014; Marrale et al., 2016; Price et al., 2017; Lin et al., 2018; Huber et al., 2019; Zhu et al., 2021) and has led to the development of extensions of DKI that provide inferences about specific aspects of the microstructure (Fieremans et al., 2011; Jespersen, 2018; Novikov et al., 2018b; Henriques et al., 2019). In addition, DKI provides information about the diffusion orientation distribution function (dODF) within a voxel, that can be used for tractography (Lazar et al., 2008; Jensen et al., 2014; Glenn et al., 2015a, 2016; Henriques et al., 2015).

Despite the utility of DKI, the use of these analysis methods depends on the availability of software implementations that work with different DKI acquisition schemes. Moreover, providing well-documented and well-tested open-source implementations of DKI could propel the development of more robust DKI reconstruction routines, novel DKI model extensions, and integration with other imaging techniques. The present paper discusses the implementation of the DKI model in the DIPY project. DIPY is an open-source software library that provides implementations of many different methods for analysis of dMRI data (Garyfallidis et al., 2014). The library, implemented in the Python programming language, relies on the robust ecosystem of scientific computing tools in Python (Perez et al., 2011). It has been in continuous development since 2009, and provides a wide array of computational neuroanatomy methods. In particular, the library provides a uniform programming interface to many different dMRI signal reconstruction models and models for inferring microstructure. Here, we will focus on providing well-tested, well-documented open-source implementations of DKI, and derived microstructural models. In addition to the implementation details, in this paper, we will also illustrate the advantages and drawbacks of the DKI model based on numerical simulations, and demonstrate the range of functionality implemented on openly available dMRI datasets.

## 2. Methods

### 2.1. Theory and Implementation

Because DKI is a direct extension of the DTI model, we begin with a brief explanation of DTI and establish our notation based on this explanation. The implementation is part of the DIPY source-code available at https://github.com/dipy/dipy . DIPY depends only on the Numpy (Harris et al., 2020), Scipy (Virtanen et al., 2020), and Nibabel (Brett et al., 2020) software libraries, with some performance bottlenecks accelerated through use of the Cython (Behnel et al., 2010) transpiler. In addition, Jupyter notebooks to generate all the results presented in this paper are available at https://github.com/dipy/dipy-dki-paper.

#### 2.1.1. Diffusion Tensor Imaging

The diffusion tensor imaging (DTI) model describes the dMRI signal *S*(**n**, *b*) using a 2nd order diffusion tensor (Basser et al., 1994). In Einstein's summation convention, the DTI model can be expressed as:

(1) $$\begin{array}{l} S\left(\text{n},b\right)=S_{0}e^{-bn_{i}n_{j}D_{ij}} \end{array}$$

where **n** is the direction of the diffusion gradient **n** = [*n*_1_, *n*_2_, *n*_3_], *b* is a value that summarizes the intensity of diffusion weighting (Le Bihan et al., 1986), **D** is the *diffusion tensor*.

To solve for **D**, it is usual to normalize the direction- and b-value-specific signal by the non-diffusion-weighted signal and re-represent this ratio in the log domain:

(2) $$\begin{array}{l} log\left(\frac{S\left(\text{n},b\right)}{S_{0}}\right)=-bn_{i}n_{j}D_{ij} \end{array}$$

This equation can be described through a set of linear equations and solved for the six independent parameters in **D** (Basser et al., 1994). In DIPY, the default fitting approach is based on a conventional weighted-least squares technique (WLS) (Chung et al., 2006). Other DTI fitting techniques are available in DIPY such as ordinary least squares (OLS), non-linear least square (NLS) and the robust estimation of tensors by outlier rejection (RESTORE) technique (Jones and Basser, 2004; Chang et al., 2005). For more information on how to fit DTI using DIPY see section 2.1.8 below

#### 2.1.2. Diffusion Tensor Metrics

After DTI fitting, the tensor **D** can be decomposed into three eigenvectors (**e**_1_, **e**_2_, and **e**_3_ and their respective eigenvalues (λ_1_ ≥ λ_2_ ≥ λ_3_) (Basser and Pierpaoli, 1996). These eigenvalues are used to compute rotationally-invariant metrics (i.e., measurements that are independent of the applied gradient direction). For instance, the mean, radial and axial diffusivities can be computed as *MD* = (λ_1_ + λ_2_ + λ_3_)/3, *RD* = (λ_2_ + λ_3_)/2, and *AD* = λ_1_. The eigenvalues of the diffusion tensor can also be used to produce measures of the degree of diffusion anisotropy (Basser and Pierpaoli, 1996). One of the most used diffusion anisotropy measures is the fractional anisotropy which is defined as Basser and Pierpaoli (1996) and Glenn et al. (2015b):

(3) $$\begin{array}{l} FA\equiv \sqrt{\frac{3}{2}}\frac{||\text{D}-MD{\text{I}}^{\left(2\right)}{||}_{F}}{||\text{D}{||}_{F}}\\ \;=\sqrt{\frac{3}{2}\frac{{\left(\lambda_{1}-MD\right)}^{2}+{\left(\lambda_{2}-MD\right)}^{2}+{\left(\lambda_{3}-MD\right)}^{2}}{\lambda_{1}^{2}+\lambda_{2}^{2}+\lambda_{3}^{2}}} \end{array}$$

where **I**^(2)^ is the fully symmetric rank-2 isotropic tensor and ||...||_*F*_ is the Frobenius norm of a tensor with rank *N* (Glenn et al., 2015b). The factor $\frac{3}{2}$ is introduced so that FA values range between 0 and 1 (from lower to higher degrees of anisotropy).

#### 2.1.3. Diffusional Kurtosis Imaging

To extend DTI and account for the excess diffusional kurtosis, DKI models the diffusional kurtosis tensor **W** in addition to the diffusion tensor **D** (Jensen et al., 2005). The DKI model can be derived by expanding the cumulants of the diffusion-weighted signal up to the 2nd order in b (Jensen et al., 2005). Using the same notation and conventions as in Equation (2):

(4) $$\begin{array}{l} log\left(\frac{S\left(\text{n},b\right)}{S_{0}}\right)=-bn_{i}n_{j}D_{ij}+\frac{1}{6}b^{2}{\left(MD\right)}^{2}n_{i}n_{j}n_{k}n_{l}W_{ijkl} \end{array}$$

Similar to DTI, the DKI model can also be described through a set of linear equations and solved for the six independent parameters of **D** and fifteen independent parameters of **W**, noting that **W** is axially symmetric (Lu et al., 2006; Tabesh et al., 2011). In addition to 15 different gradient directions to resolve the anisotropic information of **W**, the DKI model requires at least three b-values (these can include signals for *b*=0 in addition to two non-zero b-values). In DIPY, the default DKI fitting was implemented based on a weighted-least squares (WLS) technique in which weights are defined from previous diffusion parameter estimates (Veraart et al., 2013). This fitting approach was shown to provide diffusion and kurtosis estimates with higher reliability when compared to other linear least square fitting strategies and provides faster fits when compared to non-linear least square approaches. Additionally, the fitting approaches implemented in DTI (OLS, NLS, RESTORE) were adapted to the DKI model and can also be used in DIPY as alternative DKI fitting strategies (c.f. section 2.1.8 below).

#### 2.1.4. Kurtosis Tensor Metrics

Since it also fits the diffusion tensor, DKI can be used to estimate all DTI metrics (e.g., MD, RD, AD, FA explained above). Additionally, rotationally-invariant measures can be defined from the kurtosis tensor. In analogy to the definition of MD, the mean kurtosis (MK) is defined as the average of directional kurtosis coefficients across all spatial directions, which can be formulated by the following surface integral (Jensen and Helpern, 2010):

(5) $$\begin{array}{l} MK\equiv \frac{1}{4\pi }\int d\Omega_{\text{n}}K\left(\text{n}\right) \end{array}$$

where *K*(**n**) is the directional kurtosis for a given direction **n**, which can be sampled from the fitted diffusion and kurtosis tensors as:

(6) $$\begin{array}{l} K\left(\text{n}\right)=\frac{MD^{2}}{D{\left(\text{n}\right)}^{2}}n_{i}n_{j}n_{k}n_{l}W_{ijkl} \end{array}$$

with

(7) $$\begin{array}{l} D\left(\text{n}\right)=n_{i}n_{j}D_{ij} \end{array}$$

In DIPY, two approaches were implemented to compute MK:

1. the integral of Equation (5) is numerically resolved by averaging directional kurtosis values sampled for a finite number of directions. Biases of discrete direction samples can be avoided by using a spherical t-design as shown by Hardin and Sloane (1996). For the DIPY implementation of MK, a t-design of 45 directions is used.
2. The second approach is based on the analytical solution of Equation (5) (Tabesh et al., 2011), avoiding the use of discrete directional samples. This approach requires the following computing steps: (a) the rotation of the DKI tensors to a frame of reference in which **D** eigenvectors are aligned to the Cartesian axis *x*, *y* and *z*. This rotated kurtosis tensor is denoted as $\tilde{\text{W}}$; (b) the evaluation of Carlson's elliptic integrals (Carlson, 1995); and (c) the treatment of the solution's singularities for λ_1_ = λ_2_, λ_1_ = λ_3_, λ_2_ = λ_3_, and λ_1_ = λ_2_ = λ_3_. These steps were vectorized for optimal processing speed.

A comparison of these two approaches is presented in supplementary notebooks available at https://github.com/dipy/dipy-dki-paper.

Since the directional kurtosis coefficient for a given direction **n** depends on both diffusion and kurtosis tensors (Equation 6), MK as defined by Equation (5) depends on both diffusion and kurtosis tensors. To have a mean kurtosis metric independent to the diffusion tensor, the mean kurtosis tensor (MKT) is defined as Hansen et al. (2013) and Hansen and Jespersen (2017):

(8) $$\begin{array}{l} MKT\equiv \frac{1}{4\pi }\int d\Omega_{\text{n}}n_{i}n_{j}n_{k}n_{l}W_{ijkl} \end{array}$$

This latter quantity can be directly computed from the trace of the kurtosis tensor:

(9) $$\begin{array}{l} MKT=\frac{1}{5}Tr\left(\text{W}\right)=\\ \frac{1}{5}\left(W_{1111}+W_{2222}+W_{3333}+2W_{1122}+2W_{1133}+2W_{2233}\right) \end{array}$$

For voxels containing well-aligned structures, the **radial kurtosis** is defined as the average of the directional kurtosis across all directions perpendicular to the main direction of fibers which should correspond to the diffusion tensor main direction **e**_1_) (Jensen and Helpern, 2010; Tabesh et al., 2011):

(10) $$\begin{array}{l} RK\equiv \frac{1}{2\pi }\int d\Omega_{\theta }K\left(\theta \right)\delta \left(\theta \cdot {\text{e}}_{1}\right) \end{array}$$

Similar to the estimation of MK, DIPY provides two methods to compute RK based on a numerical and an analytical approach:

1. Equation (10) can be numerically computed by averaging directional kurtosis values for directions perpendicular to **e**_1_. The directional kurtosis values can be sampled from the fitted kurtosis tensor using equation (6). Directions **θ** that are evenly sampled and perpendicular to **e**_1_ ^1^.
2. Alternatively, Equation (4) can be solved analytically, avoiding discrete perpendicular direction samples (Tabesh et al., 2011). This approach requires the rotation of the kurtosis tensor and the treatment of a singularity for λ_2_ = λ_3_.

The axial kurtosis is defined as the directional kurtosis along the main direction of well-aligned structures:

(11) $$\begin{array}{l} AK\equiv K\left({\text{e}}_{1}\right) \end{array}$$

This quantity can be computed from one of the following:

1. The directional kurtosis coefficient along the tensor eigenvector (i.e., applying **e**_1_ into Equation (6);
2. From the tensor element ${\tilde{W}}_{1111}$ of the rotated kurtosis tensor (i.e., $AK=MD^{2}/\lambda_{1}^{2}{\tilde{W}}_{1111}$, Tabesh et al., 2011).

Although both approaches lead to the exact calculation of AK, the former and latter estimators will be referred to as the numerical and analytical methods, respectively, to keep the nomenclature consistent to the estimation strategies of MK and RK.

Similar to the definition of FA for the diffusion tensor, the anisotropy of the kurtosis tensor can be quantified as Glenn et al. (2015b):

(12) $$\begin{array}{l} KFA\equiv \frac{||\text{W}-MKT{\text{I}}^{\left(4\right)}|{|}_{F}}{||\text{W}|{|}_{F}} \end{array}$$

where **I**^(4)^ is the fully symmetric rank 4 isotropic tensor, ||...||_*F*_ is the Frobenius norm (Glenn et al., 2015b), and MKT is the mean kurtosis tensor defined by Equation (9). Analogs to the FA of the diffusion tensor, KFA quantifies lower to higher kurtosis tensor anisotropy in a range between 0 and 1.

#### 2.1.5. White Matter Tract Integrity Model

One way to interpret the information captured by DKI is to fit additional microstructural models to the diffusion and kurtosis tensors (Jensen et al., 2005; Jensen and Helpern, 2010; Fieremans et al., 2011; Jespersen, 2018; Novikov et al., 2018b). This approach provides DKI-derived scalar quantities that are potentially more specific to microstructural properties of the tissue, such as the fraction of signal contributions due to extra- or inter-cellular spaces. However, as in the case of microstructural models applied directly to dMRI signals (Assaf and Basser, 2005; Jespersen et al., 2007; Zhang et al., 2012; Kaden et al., 2016b), the interpretation of these quantities is only valid if the assumptions of the microstructural models are met (Lampinen et al., 2017, 2019; Novikov et al., 2018a; Henriques et al., 2019). The White Matter Tract Integity (WMTI ) model (Fieremans et al., 2011, 2013) relates the diffusion and kurtosis tensors to the parameters of a two compartments model representing the intra- and extra-cellular components of aligned white matter fibres:

(13) $$\begin{array}{l} S\left(\text{n},b\right)/S_{0}=f^{ia}e^{-bn_{i}n_{j}D_{ij}^{ia}}+\left(1-f_{ia}\right)e^{-bn_{i}n_{j}D_{ij}^{ea}} \end{array}$$

where *f*^*ia*^ is the intra-axonal water fraction (AWF), **D**^*ia*^ is the intra-axonal diffusion tensor and **D**^*ea*^ is the extra-axonal diffusion tensor.

The WMTI model relies on the following assumptions:

1. The tissue is only described by non-exchanging intra- and extra-cellular compartments. Other signal components, such as from glia cell, have to be in fast exchange with the extra-cellular compartment.
2. The intra-cellular diameter of axons is much smaller than the volume probed by diffusing particles. That is, intra-cellular RD is practically zero.
3. In all directions water-molecules can more freely move in the extra-cellular volume. That is, intra-cellular AD is smaller than extra-cellular AD
4. Intra-cellular spaces are well-aligned to each other. This does not apply to voxels containing fiber dispersion or crossing.
5. Effects of the interaction of water molecules with the boundaries of different intra- and extra-cellular compartments (e.g., collision with cell membranes or myelin sheaths) or with macromolecules are negligible.

Despite several studies that have demonstrated that these assumptions are unlikely to hold in many cases (e.g., Dhital et al., 2018), WMTI measures were still shown useful as sensitive biomarkers for the characterization of progression of white matter microstructural alterations in health and disease (e.g., Hui et al., 2012; Fieremans et al., 2013).

In DIPY, WMTI is implemented as follows:

1. Computing the maximum directional kurtosis. Kurtosis is evaluated using Equation (6) for 100 uniformly distributed directions **n** and direction of the maximal value is used to seed a quasi-Newton method algorithm to optimize the following problem:

(14) $$\begin{array}{l} K_{max}=\begin{array}{c} max\\ \theta \phi \end{array}\left[K\left(n\right)\right] \end{array}$$

where θ and ϕ are the polar and azimuth coordinates of the unit direction ***n*** that maximizes the kurtosis.

2. Computing the axonal water fraction. For a system described by Equation (7), the maximum kurtosis is expected to be perpendicular to the main direction (Fieremans et al., 2011; Henriques et al., 2015). Under the assumption that intra-cellular RD is zero, the axonal volume fraction (AWF) is computed as Jensen and Helpern (2010), Fieremans et al. (2011), and Jespersen (2018):

(15) $$\begin{array}{l} AWF=\frac{K_{max}}{K_{max}+3} \end{array}$$

3. Decoupling the compartmental diffusivities. Assuming that extra-axonal diffusivity is always higher than the intra-axonal diffusivity, the directional diffusivities for both intra- and extra-cellular compartments *D*(**n**)_*i*_ and *D*(**n**)_*e*_ are estimated for given directions **n**, using the following expressions:

(16) $$\begin{array}{l} D{\left(n\right)}_{i}=D(n)\left[1-\sqrt{\frac{K(n)(1-AWF))}{3AWF}}\right] \end{array}$$

and

(17) $$\begin{array}{l} D{\left(\text{n}\right)}_{e}=D\left(\text{n}\right)\left[1+\sqrt{\frac{K\left(\text{n}\right)AWF}{3\left(1-AWF\right)}}\right] \end{array}$$

where *D*(**n**) and *K*(**n**) are computed from Equations (7, 6). The tensors **D**^*ia*^ and **D**^*ea*^ are computed from *D*(**n**)_*i*_ and *D*(**n**)_*e*_ samples for at least six different directions **n** (Fieremans et al., 2013).

4. Deriving WMTI metrics. In addition to the AWF, other WMTI metrics are defined from tensors **D**^*ia*^ and **D**^*ea*^: the axonal diffusivity *D*^*ia*^ defined as the trace of **D**^*ia*^; the axial and radial diffusivities of the extra-cellular diffusion tensor *AD*^*ea*^ and *RD*^*ea*^; and the extra-cellular tortuosity which is defined as the ratio between *AD*^*ea*^ and *RD*^*ea*^.

#### 2.1.6. Mean Signal Diffusional Kurtosis Imaging

The DKI model aims to characterize the full 3D directional dependence of diffusional kurtosis, which is influenced by tissue microstructural properties. For example, by the sizes of different compartments, their apparent diffusivities, and volume fractions. However, directional kurtosis is also affected by tissue organization: the degree of dispersion, crossing or fanning (Henriques et al., 2015). Increased specificity toward microstructural properties can be achieved by measuring a scalar excess-kurtosis index from powder-averaged signals (Henriques, 2018; Henriques et al., 2019). That is, averaged signals across evenly-sampled gradient direction for each b-value that are independent of the tissue orientation distribution function (Jespersen et al., 2013; Kaden et al., 2016b). In DIPY, this technique is referred to as the mean signal diffusional kurtosis imaging (MSDKI). Analogs to the derivation of DKI from the directional diffusion-weighted signals, the MSDKI model can be derived from powder-averaged signals $\bar{S}\left(b\right)$ using the second-order cumulant expansion (Henriques, 2018; Henriques et al., 2019):

(18) $$\begin{array}{l} log\left(\frac{\bar{S}\left(b\right)}{S_{0}}\right)=-bD_{p}+\frac{1}{6}b^{2}{\left(D_{p}\right)}^{2}K_{p} \end{array}$$

where *D*_*p*_ and *K*_*p*_ are the diffusivity and excess-kurtosis of the powder-average signals. In DIPY, these quantities are referred to as the mean signal diffusivity (MSD) and mean signal kurtosis (MSK). It is important to note that, while MSD theoretically is equal to the standard mean diffusivity (MD) (Henriques et al., 2019), MSK is equal to mean kurtosis tensor (MKT) subtracted by a mesoscopic dispersion correction factor Ψ which can be calculated from the diffusion tensor (Henriques et al., 2020a), i.e.,:

(19) $$\begin{array}{l} MSK=MKT-\text{Ψ} \end{array}$$

with

(20) $$\begin{array}{l} \text{Ψ}=\frac{6}{5}-\frac{2}{5}\frac{D_{11}^{2}+D_{22}^{2}+D_{33}^{2}+2D_{12}^{2}+2D_{13}^{2}+2D_{23}^{2}}{MD^{2}} \end{array}$$

Diffusion-weighted data can be acquired with different numbers of gradient directions *N*_*g*_ for different b-values. Therefore, in DIPY, the MSDKI model (Equation 18) is fitted using a weighted least square approach in which weights for each b-value are set to $w=N_{g}\bar{S}\left(b\right)$ (Henriques, 2018).

#### 2.1.7. MSDKI-Based Microstructural Models

Analogs to the DKI metrics, the parameters of MSDKI can also be related to microstructural models. For instance, Henriques et al. (2019) showed that MSDKI captures the same information than the spherical mean technique (SMT) microstructural models (Kaden et al., 2016a,b). In this way, the SMT model parameters can be directly computed from MSDKI. In DIPY, the intrinsic diffusivity (*D*_*I*_) and the axonal water fractions (AWF) of the two-compartmental SMT model parameters (Kaden et al., 2016a) can be estimated from MSDKI parameters was implemented by inverting the following equations (Henriques et al., 2019):

(21) $$\begin{array}{l} MSD=\frac{D_{I}\left(1+2{\left(1-AWF\right)}^{2}\right)}{3} \end{array}$$

and

(22) $$\begin{array}{l} MSK=\frac{216AWF-504AWF^{2}+504AWF^{3}-180AWF^{4}}{135-360AWF+420AWF^{2}-240AWF^{3}+60AWF^{4}} \end{array}$$

Although SMT models are general to any tissue configuration (i.e., general to well-aligned, crossing or dispersing fibers), the two-compartmental model assumes that: (1) the axial diffusivities of both intra- and extra-cellular spaces are equal to the in the intrinsic diffusivity (*D*_*I*_), (2) the extra-cellular radial diffusivity follows the first order tortuosity assumption (*RD* = (1−*AWF*)*D*_*I*_); and (3) the intra-cellular radial diffusivity is zero.

#### 2.1.8. Software Implementation

DIPY uses object-oriented design for defining and fitting diffusion models. A class hierarchy for diffusion reconstruction models provides a uniform application programming interface (API) for defining and fitting these models. We follow a pattern established in the scikit learn machine learning library (Pedregosa et al., 2011) whereby a model object is first defined based on the gradient table containing the information of the diffusion acquisition parameters (see Garyfallidis et al., 2014 for details and also code examples in the present paper's jupyter notebooks https://github.com/dipy/dipy-dki-paper). For DTI, the tensor model class instance can be imported and initialized in the following way (Garyfallidis et al., 2014):



  from dipy.reconst import dti
  model = dti.TensorModel(gtab,
  fit_method='WLS')


Note that the optional input parameter fit_method sets the DTI fitting method—additionally to the default WLS fitting strategy, DTI can also be fitted using the OLS, NLS, and RESTORE approaches (c.f. section 2.1.1 "Diffusion Tensor Imaging"). After importing and initializing DTI model, its model fitting is then done separately once data is available:



      dtifit = model.fit(data, mask=mask)

where data is a numpy array class instance containing the diffusion-weighted data (last dimension of this matrix has to correspond to the b-values and b-vectors) and the optional input parameter mask can be used to indicate which voxels should be processed (this option may be useful to avoid unnecessary calculations on the background of the image).

This sets the model parameters as attributes of the fit object. Calculation of scalar quantities, such as FA, is deferred until these are needed. However, once these properties are called, they are immediately set as attributes of their object (a pattern called “one-time property” (Rokem et al., 2009). This simplifies access to the these scalars. For example, to access the standard DTI metrics mentioned in section 2.1.2, the following command lines are used:



      MD = dtifit.md
      AD = dtifit.ad
      RD = dtifit.rd
      FA = dtifit.fa

In analogy to DTI, fitting the DKI model is:



      from dipy.reconst import dki
      model = dki.DiffusionKurtosisModel(gtab,
      fit_method=‘‘WLS'')
      dkifit = model.fit(data, mask=mask)

Since DKI was implemented from inheritance of the DTI implementations, these can be used to extract the diffusion tensor metrics (section 2.1.2), in addition to the kurtosis tensor metrics (section 2.1.4):



      MD = dkifit.md
      AD = dkifit.ad
      RD = dkifit.rd
      FA = dkifit.fa
      MK = dkifit.mk()
      MKT = dkifit.mkt
      AK = dkifit.ak()
      RK = dkifit.rk()
      KFA = dkifit.kfa

Note that dkifit.mk, dkifit.ak, and dkifit.rk are not implemented as a one-time property but as class function to allow users to select the metrics calculation strategy (numerical vs. analytical solution) as well as define the ranges of plausible kurtosis values (c.f. section 2.1.4 and jupyter notebooks).

Regarding the WMTI model, its model class instance can be imported, initialized and fitted in the following way:



      from dipy.reconst import dki_micro
      model = dki_micro.KurtosisMicrostructure
      Model(gtab)
      wmtifit = model.fit(data, mask=mask)

The WMTI model parameters (AWF, *D*^*ia*^, *AD*^*ea*^, *RD*^*ea*^, and the extra-cellular tortuosity) are obtained using the respective class methods/attributes:



      AWF = wmtifit.awf
      ADia = wmtifit.axonal_diffusivity
      ADea = wmtifit.hindered_ad
      RDea = wmtifit.hindered_rd
      TORT = wmtifit.tortuosity

The mean signal diffusion kurtosis imaging (section 2.1.6) and its respective SMT model conversion (section 2.1.7) can be processed using the following lines:



      from dipy.reconst import msdki
      model = msdki.MeanDiffusionKurtosis
      Model(gtab)
      msdkifit = model.fit(data, mask=mask)
  
      MSD = msdkifit.msd
      MSK = msdkifit.msk
      AWF = msdkifit.awf
      DI = msdkifit.di

#### 2.1.9. Numerical Simulations for DKI Unit Testing

DIPY uses both rigorous unit testing (with pytest) and continuous integration (Travis, Appveyor and Azure Pipelines) to validate software implementations against known analytically-derived cases, and to assess any change to the software with fixes and enhancements that are introduced. In this work, we ensured that all the code statements in DIPY's DKI implementations are exercised in automated testing, i.e., there is 100% test coverage (Zhu et al., 1997).

To test our DKI implementations, numerical simulations were produced for a sum of *N* Gaussian diffusion compartments (i.e., effects of interaction between diffusing water molecules and compartment's obstacles are assumed to be negligible):

(23) $$\begin{array}{l} S\left(\text{n},b\right)/S_{0}=\overset{N}{\underset{m=1}{\sum }}f^{m}e^{-bn_{i}n_{j}D_{ij}^{m}} \end{array}$$

where *f*^*m*^ is the apparent water fraction of a tissue compartment *m*, and $D_{ij}^{m}$ are the elements of its Gaussian diffusion. The ground truth of the elements of the total diffusion tensor *D*_*ij*_ and total kurtosis tensor *W*_*ijkl*_ of the multiple compartment system are computed as Lazar et al. (2008), Jensen and Helpern (2010), and Henriques et al. (2015):

(24) $$\begin{array}{l} D_{ij}=\overset{N}{\underset{m=1}{\sum }}f^{m}D_{ij}^{m} \end{array}$$

and

(25) $$\begin{array}{l} W_{ijkl}=\frac{1}{MD^{2}}(\sum_{m=1}^{N}f^{m}\left[D_{ij}^{m}D_{kl}^{m}+D_{ik}^{m}D_{jl}^{m}+D_{il}^{m}D_{jl}^{m}\right]\\ \;-D_{ij}D_{kl}-D_{ik}D_{jl}-D_{il}D_{jl}) \end{array}$$

To remove the effects of the cumulants of Equation (23) associated with terms higher than the 2nd order in b, synthetic diffusion-weighted signals for implementation testing are produced by plugging the ground truth **D** and **W** tensors into Equation (4). These synthetic signals were produced for different ground truth compartmental tensors **D**^(*m*)^ (with different axial and radial diffusivities **AD**^(*m*)^ and **RD**^(*m*)^ and different orientations), so that different diffusion metrics are validated for different simulation scenarios. A summary of different sets of ground truth parameters and the different checks used for DKI unit testing are presented in Table 1. In addition to these, the Carlson integrals were also evaluated according to the numerical checks suggested in the original work by Carlson (Carlson, 1995).

**Table 1:** Details of numerical simulations used for DKI Unit Testing Numerical-a brief description of each simulations, the simulation compartmental diffusion parameters (where *AD*^(*m*)^, *RD*^(*m*)^, *f*^(*m*)^, ϕ^(*m*)^, and θ^(*m*)^ are the axial diffusivity, radial diffusivity, water fraction, polar orientation angle and azimuthal orientation angle of a given compartment *m*), and the checks preformed are presented on the left, middle and right columns, respectively.

**Simulations**	**G.T. parameters**	**Tests**
Isotropic tensors (2 compartments)	*AD*^(1)^ = *RD*^(1)^ = 0.99 *f*^(1)^ = 0.5	1) Check if MK, RK, AK, MKT, MSK, and directional kurtosis samples are equal to the G.T. isotropic kurtosis value of 0.4581;
	*AD*^(2)^ = *RD*^(2)^ = 2.26 *f*^(2)^ = 0.5	2) Check that KFA = 0;
		3) Check that the maximum kurtosis calculation procedure does not generate an error (note that isotropic kurtosis does not have a maximum);
Single fiber (2 compartments)	*AD*^(1)^ = 0.99, *RD*^(1)^ = 0, *f*^(1)^ = 0.49	1) Check if rotated Kurtosis tensor $\tilde{\text{W}}$ have only the following non-zero elements: ${\tilde{W}}_{1111}=1.7067$, ${\tilde{W}}_{2222}={\tilde{W}}_{3333}=0.800$, ${\tilde{W}}_{1122}={\tilde{W}}_{1133}=0.3897$, ${\tilde{W}}_{2233}=0.2670$
	*AD*^(2)^ = 2.26, *RD*^(2)^ = 0.87, *f*^(2)^ = 0.51	2) Test if the direction of maximum kurtosis is perpendicular to the main fiber direction Henriques et al. (2015);
	θ^1^ = θ^2^ = *rand* ϕ^1^ = ϕ^2^ = *rand*	3) Test if the value of maximum apparent kurtosis is equal to RK;
Henriques et al. (2015)		4) Test if WMTI fitted parameters matches their G.T. values
Crossing fibers (4 compartments = 2 intra-cellular + 2 extra-cellular)	*AD*^(1)^ = *AD*^(3)^ = 0.99 *RD*^(1)^ = *RD*^(3)^ = 0 *f*^(1)^ = *f*^(3)^ = 0.245	1) Check if estimated *D*_*ij*_ and *W*_*ijkl*_ elements for all DKI fitting procedures (OLS, WLS, NLS, RESTORE) match the ground truth parameters given by Equations (23, 24);
	*AD*^(2)^ = *AD*^(4)^ = 2.23 *RD*^(2)^ = *RD*^(4)^ = 0.87 *f*^(2)^ = *f*^(4)^ = 0.255	2) Check if rotated Kurtosis tensor $\tilde{\text{W}}$ have the same values of a reference **W** generated for a scenario in which the **D** is already aligned to the cartesian axis *x*, *y*, and *z*;
	θ^1^ = θ^2^ = 80^o^ ϕ^1^ = ϕ^2^ = 10^o^	3) Check if MK, AK, and RK numerical solutions match their analytical solutions;
	θ^3^ = θ^4^ = 20^o^ ϕ^3^ = ϕ^4^ = 30^o^	4) Test if the direction of maximum apparent kurtosis is perpendicular to both fibers Henriques et al. (2015)
Fibers crossing at 90^o^ (4 compartments= 2 intra-cellular + 2 extra-cellular)	(same diffusivities as above) θ^1^ = θ^2^ = 90^o^ ϕ^1^ = ϕ^2^ = 0^o^ θ^3^ = θ^4^ = 20^o^ ϕ^3^ = ϕ^4^ = 30^o^	1) Check if MK matches the values of the previous simulation (Note that fibers crossing at 90^o^ has a prolate diffusion tensor which corresponds to MK analytical solution singularity)
Two crossing compartments	*AD*^(1)^ = *AD*^(2)^ = 1.7 *RD*^(1)^ = *RD*^(2)^ = 0.3 *f*^(1)^ = *f*^(2)^ = 0.5	1) Check if KFA for this scenario is equal to $\sqrt{\left(13/5\right)}$ (Glenn et al., 2015b)

### 2.2. Simulated Experiments

**Figure 1:**
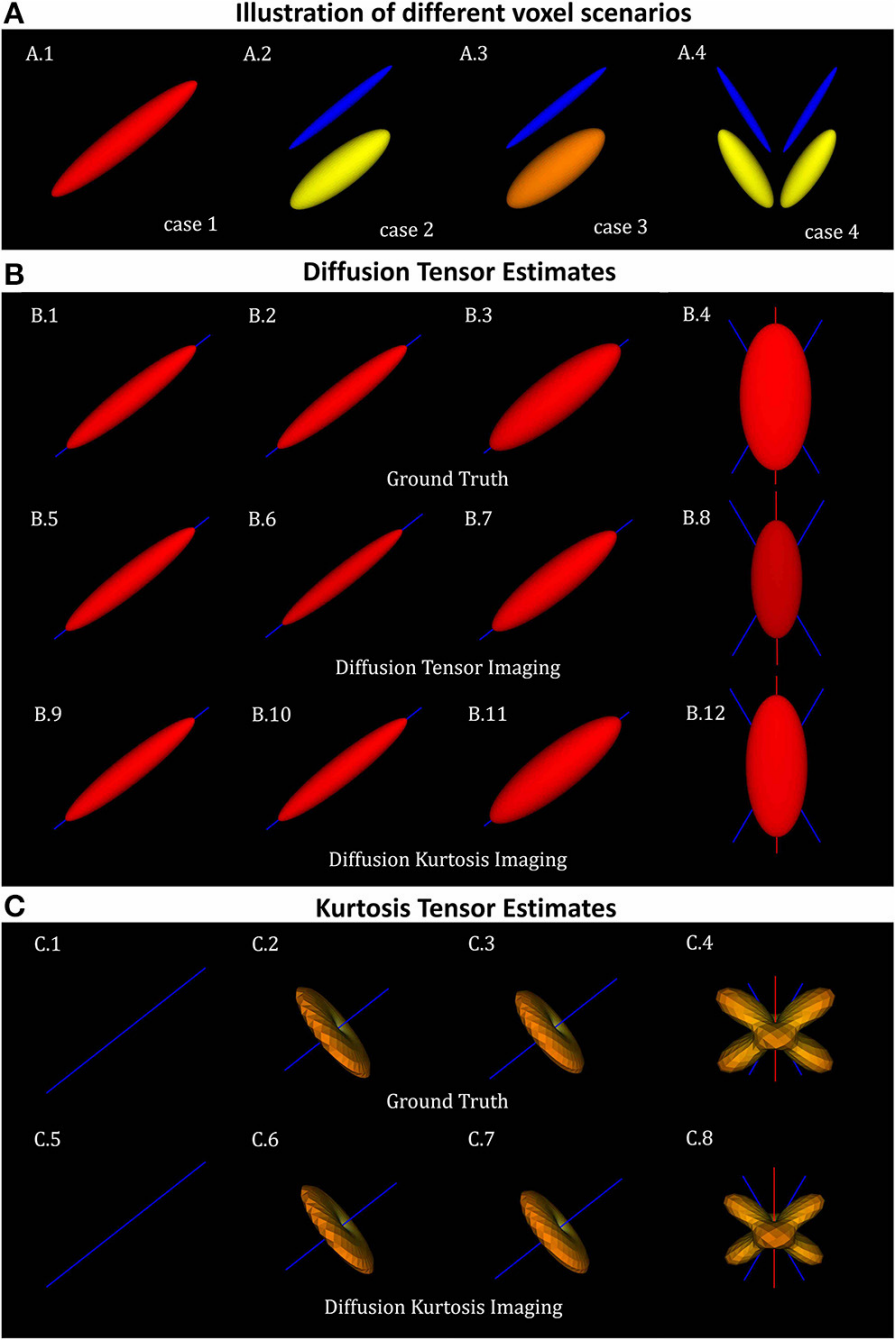
Diffusion and kurtosis tensors from single and multi-tensor toy-models. **(A)** Illustration of the tensor components of each simulation case: (A.1) single tensor with *AD* and *RD* = 1.7 × 10^−3^*mm*^2^/*s* and 1.3 × 10^−3^*mm*^2^/*s*; (A.2) mixture of collinear tensors with *AD*_1_, *RD*_1_, *AD*_2_, and *RD*_2_ = 1.4 × 10^−3^, 0.1 × 10^−3^, 2 × 10^−3^, 0.5 × 10^−3^*mm*^2^/*s* (toy-model of a healthy fiber population); (A.3) mixture of collinear tensors with *AD*_1_, *RD*_1_, *AD*_2_, and *RD*_2_ = 1 × 10^−3^, 0.1 × 10^−3^, 2 × 10^−3^, 0.7 × 10^−3^*mm*^2^/*s* (toy-model of a damaged fiber population); and (A.4) mixture of crossing tensors (toy-model of crossing healthy fiber populations). **(B)** Diffusion tensors for each voxel simulation: (B.1–B.4) Ground truth diffusion tensors; (B.5–B.8) diffusion tensors computed from DTI fit; and (B.9–B.12) diffusion tensors computed from DKI. **(C)** kurtosis tensors for each voxel simulations: (C.1–C.4) Ground truth kurtosis tensors; (C.5–C.8) Kurtosis tensors fitted by DKI. In this figure, diffusion tensor are plotted in their ellipsoid representation, while kurtosis tensors are plotted as the 3D spatial variation of apparent kurtosis coefficients.

To illustrate the accuracy of DKI in different scenarios of its different metrics, diffusion tensors and kurtosis tensors are first processed on single voxel synthetic signal (Equation 23) and compared to the ground truth tensors (Equations 24, 25) for four different sets of ground truth parameters (Figure 1A):

1. Single axial symmetric diffusion tensor component with axial and radial diffusivities of 1.7e-3 and 0.3e-3 *mm*^2^/*s*. These diffusivities were set according to typical white matter diffusion tensor estimates.
2. Two aligned axial symmetric diffusion tensor components with equal volume fractions. This scenario was produced to consider typical diffusion heterogeneity of voxels containing single healthy white matter fiber populations. As a toy-model two diffusion tensors are referred to as intra- and extra-cellular components. Axial diffusivity, radial diffusivity and volume fraction for the intra-cellular components were set to 1.4e-3 *mm*^2^/*s*, 0.1e-3 *mm*^2^/*s*, and 0.5, while the axial diffusivity, radial diffusivity and volume fraction for the extra-cellular component were set to 2e-3 *mm*^2^/*s*, 0.5e-3 *mm*^2^/*s*, and 0.5, respectively.
3. Two aligned axial symmetric diffusion tensor components with different volume fractions. This scenario was produced as a toy-model of a damaged single fibre population. For this, relative to scenario 2, the volume fraction of the intra-cellular cellular component was decreased to 0.3 while the radial diffusivity of the extra-cellular space was increased to 0.7e-3 *mm*^2^/*s*.
4. Four axial symmetric diffusion tensor components with equal volume fractions. This scenario was produced as a toy model to represent the intra- and extra-cellular contributions of two fiber populations crossing at 60 degrees.

The synthetic signals for each set of ground truth parameters are generated according to the same gradient directions **n** of the CFIN human brain dataset and b-values = 1,000, 2,000, 3,000 *s*/*mm*^2^ *(vide infra)* in addition to six b-value=0 images. After assessing the fitted tensor accuracy, the robustness of DKI metrics are tested in synthetic signals corrupted with Rician noise at different SNR levels. To assess the robustness of our DKI implementations relative to other software implementations, these simulations were also processed using the DKI procedures provided by PyDesigner (https://github.com/m-ama/PyDesigner) a recently developed software that combines functionality of the Diffusion Kurtosis Estimator (DKE) and Designer packages (Jensen et al., 2005, 2016; Jensen and Helpern, 2010; Fieremans et al., 2011; Tabesh et al., 2011; Glenn et al., 2015b; Ades-Aron et al., 2018; McKinnon et al., 2018; Moss et al., 2019; Moss and Jensen, 2021)—more information of these packages are described in discussion section 4.2. Since PyDesigner allows the introduction of parameter constraints, two version of PyDesigner estimates are tested and compare: (1) data fitting with no constraints in diffusion and kurtosis estimation; and (2) data fitting by constraining all apparent directional kurtosis to positive values.

### 2.3. MRI Experiments

Open-source software tools such as DIPY serve a particularly important role in advancing science in a period in which we are seeing an increase in availability of open datasets. In the work presented here, we use some of these open datasets to illustrate typical contrasts of different DKI metrics and to show the functionality of our DKI implementations.

#### 2.3.1. DKI-Specific Datasets

To illustrate some typical DKI contrasts, we processed two datasets that were specifically collected to support the development of DKI modeling approaches (Hansen and Jespersen, 2016a). These datasets will be referred to as the **CFIN datasets** and correspond to data of brains of two different species (human and rat) acquired using a scanner from two vendors (Siemens and Bruker Biospin). The human brain dataset was acquired *in vivo* in a Siemens 3T MRI instrument, with a 32 channel head coil. Measurements along 33 diffusion gradient directions for multiple b-values were sampled in steps of 200 *s*/*mm*^2^ from 200 *s*/*mm*^2^ to 3,000 *s*/*mm*^2^ in addition to a single acquisition for b-value=0. . This data was acquired with inversion recovery to suppress cerebrospinal fluid signal. The rat brain dataset was acquired *ex vivo* for an half brain hemisphere using a Bruker Biospec 9.4 T MRI system equipped with a 15 mm quadrature coil. Although the rat brain dataset was originally acquired for a larger number of b-values, to decrease processing time, here we only selected the 33 diffusion gradient directions/repetitions for b-values = 0, 1,000, 2,000, 3,000, 4,000, 5,000 *s*/*mm*^2^. More information about these datasets were previously reported (Hansen and Jespersen, 2016a).

Since DKI involves the estimation of a large number of parameters and since the non-Gaussian components of the diffusion signal are more sensitive to artefacts (Jensen et al., 2005; Tax et al., 2015), it might be favorable to suppress the effects of noise and artefacts before diffusional kurtosis fitting. Noise of the CFIN datasets were suppressed using the Marcenko-Pastur PCA denoising algorithm as proposed by Veraart et al. (2016b), while the impacts of Gibbs ringing artefacts were attenuated using a sub-voxel Fourier Transform shifts (Kellner et al., 2016; Henriques, 2018). Both pre-processing procedures were shown to provide optimal performances for DKI (Ades-Aron et al., 2018; Henriques, 2018). Open-source implementations of these procedures are available in DIPY.

After all data is pre-processed, DKI-based metrics are extracted using the DIPY NLS-DKI fitting (MK, AK, and RK estimates are obtained using the default analytical solution), WLS MSDKI fitting, and WMTI fitting routines. DKI parametric maps are then compared to the maps obtained using the PyDesigner software. To highlight the differences between the different procedures, this later comparison is performed for a subset of CFIN's human brain dataset. For this data subset we selected all the signals acquired for b-values = 0, 200, 1,000, 2,000, 3,000 *s*/*mm*^2^.

#### 2.3.2. Testing DKI in a Large Open Dataset

We tested the DKI implementation in data from the Human Connectome Project (HCP) (Glasser et al., 2016). The HCP has collected data about brain connectivity from 1,200 individuals and includes measurements of functional and structural MRI, as well as dMRI, in addition to many measurements of phenotypical information (e.g., behavioral assessments) (Sotiropoulos et al., 2013; Glasser et al., 2016). We used data from the 1,064 subjects for which a complete dMRI measurement was available to compare the DTI and DKI models. Briefly: the measurements conducted included 270 measurement directions, 90 directions in each of three b-value tiers: *b* ≈ 1, 000*s*/*mm*^2^, *b* ≈ 2, 000*s*/*mm*^2^ and *b* ≈ 3, 000*s*/*mm*^2^. In addition, 18 measurements with b-values close to 0 (*b* ≈ 5*s*/*mm*^2^ were taken. Voxel dimensions were 1.25 × 1.25 × 1.25 *mm*^3^. We used data that was preprocessed using the HCP preprocessing pipeline. Additional details of measurement and processing were previously published (Sotiropoulos et al., 2013). The data was accessed in the Amazon Simple Storage Web Service (S3) through the AWS Open Data program.

To assess the goodness of fits to the data, we calculated Akaike's information criterion (AIC) for DKI and DTI fits. AIC is a measure of goodness of fit that balances the residual sum of squares of a model with the number of free parameters (Cavanaugh, 1997) and it is asympotically equivalent to cross-validation (Stone, 1977). In addition to goodness of fit, we can assess the reliability of metrics derived from both models in this data. This is done by sub-sampling the data into different combinations of b-value tiers, as previously done in (Veraart et al., 2011). FA was calculated using DTI for the *b* ≈ 1, 000*s*/*mm*^2^ tier and for a combination of the *b* ≈ 1, 000*s*/*mm*^2^ tier and the *b* ≈ 2, 000*s*/*mm*^2^ tier. FA was also calculated using DKI for a combination of *b* ≈ 1, 000*s*/*mm*^2^ and *b* ≈ 2, 000*s*/*mm*^2^ and for a combination of *b* ≈ 1, 000*s*/*mm*^2^ and *b* ≈ 3, 000*s*/*mm*^2^. Reliability is assessed by computing the differences between the values of FA/MD for the two sub-samples. We assessed and compared whether these values tended to be small by looking at the median absolute deviation (MAD). In addition, we assessed whether there are systematic differences between different samples—whether these values tended to be centered around zero—by examining their median value.

## 3. Results

### 3.1. DKI Simulations

The ground truth of the diffusion tensors mixture for the four different voxel simulations is shown in the Figure 1A. For all voxel simulations, Figure 1B shows the ground truth diffusion tensor computed using (Equation 24) (Figures 1B.1–B.4), the diffusion tensors computed from DIPY's DTI fit (Figures 1B.5–B.8), and the diffusion tensors computed from DIPY's DKI fit (Figures 1B.9–B.12). Note that, for the multi-component simulations (Figures 1A.2–A.4), diffusion tensors from DKI (Figures 1B.10–B.12) are closer to the ground truths (Figures 1B.2–B.4) than the diffusion tensors from DTI (Figures 1B.6–B.8). For a visualization of the kurtosis tensors, the apparent directional kurtosis computed from both ground truth (Equation 25) and DKI-fitted tensors are shown in Figure 1C. Both ground truth and fitted kurtosis tensors are null for single diffusion component simulations (Figures 1C.1,C.5). They show maximum values perpendicular to the direction of the aligned multi-tensors (Figures 1C.2,C.3,C.6,C.7) Maximum kurtosis values are also present perpendicularly to the both directions of crossing fiber populations (Figures 1C.4,C.8).

**Figure 2:**
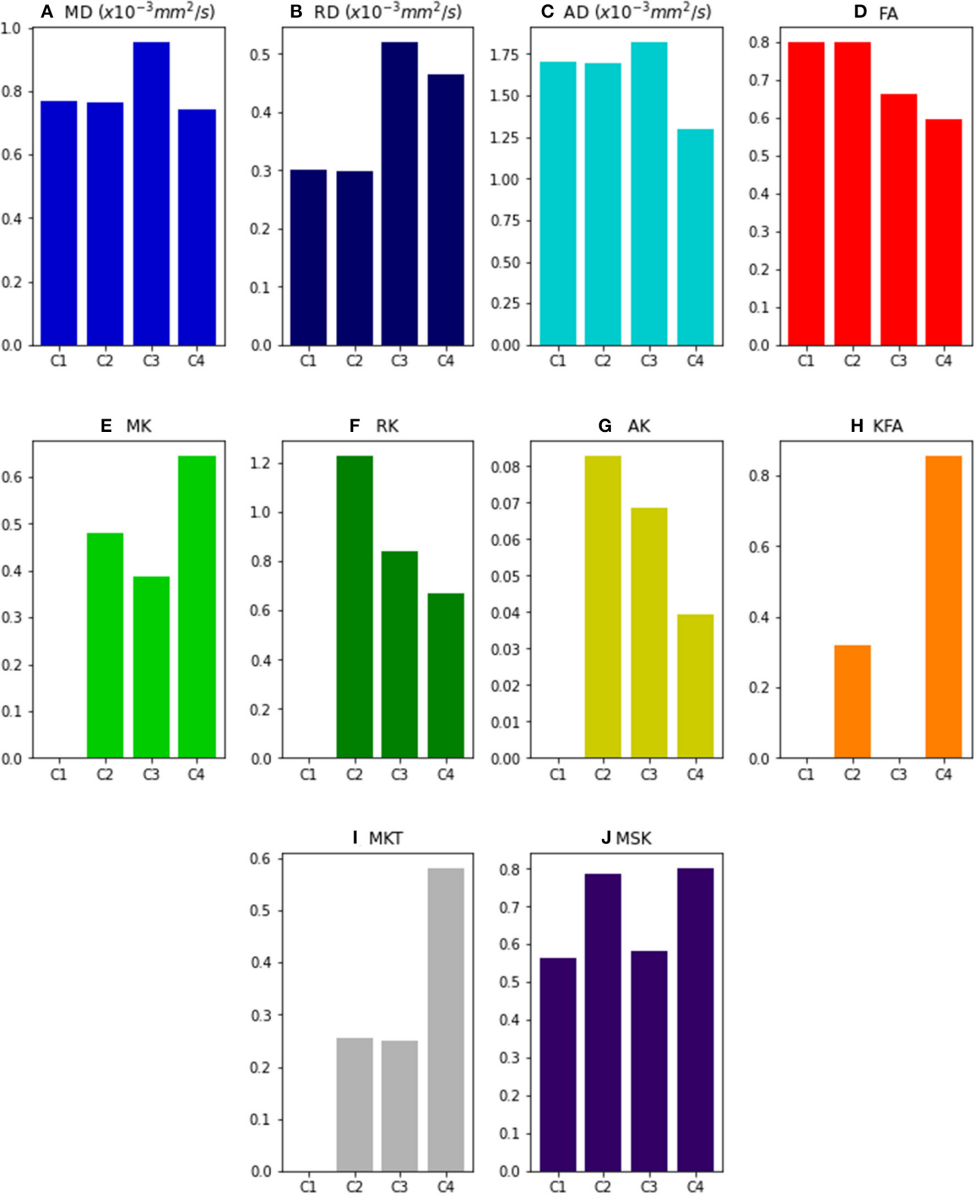
DKI and MSDKI diffusion and kurtosis metrics for the four voxel simulations: **(A)** Mean diffusivity; **(B)** Radial diffusivity; **(C)** Axial diffusivity; **(D)** Fractional Anisotropy; **(E)** mean kurtosis; **(F)** Radial Kurtosis; **(G)** Axial kurtosis; **(H)** Kurtosis Fractional Anisotropy; **(I)** Mean Kurtosis Tensor; **(J)** Mean signal kurtosis.

Figure 2 shows the values of DKI metrics obtained for the noise free synthetic signals of all four voxel simulations. Simulation cases 1 and 2 were designed to have equal values of MD, RD, AD, and FA. However, the presence of multi-tensors for voxel case 2 is revealed by the positive values of the standard kurtosis values (i.e., MK, RK, AK , MKT). MSK is positive even for synthetic signals of a single diffusion tensor (case 1) because, as being a metric from directional-averaged signals, it is sensitive to diffusion variance across different gradient directions. Relative to a toy model of a healthy fiber population (voxel case 2), all standard diffusivity metrics (MD, RD, and AD) show higher values for the toy model of a damaged fiber population (case 3), while kurtosis metrics shows lower values (except from MKT). FA shows also decreased values for the damaged fiber toy model; however, its values are even lower for the toy model of healthy crossing fibers (case 4). MSK shows equal values for simulation case 2 and 4, confirming that, in opposite of MK and MKT, MSK is only dependent on the diffusion variance across voxel components and invariant to added crossing compartments.

**Figure 3:**
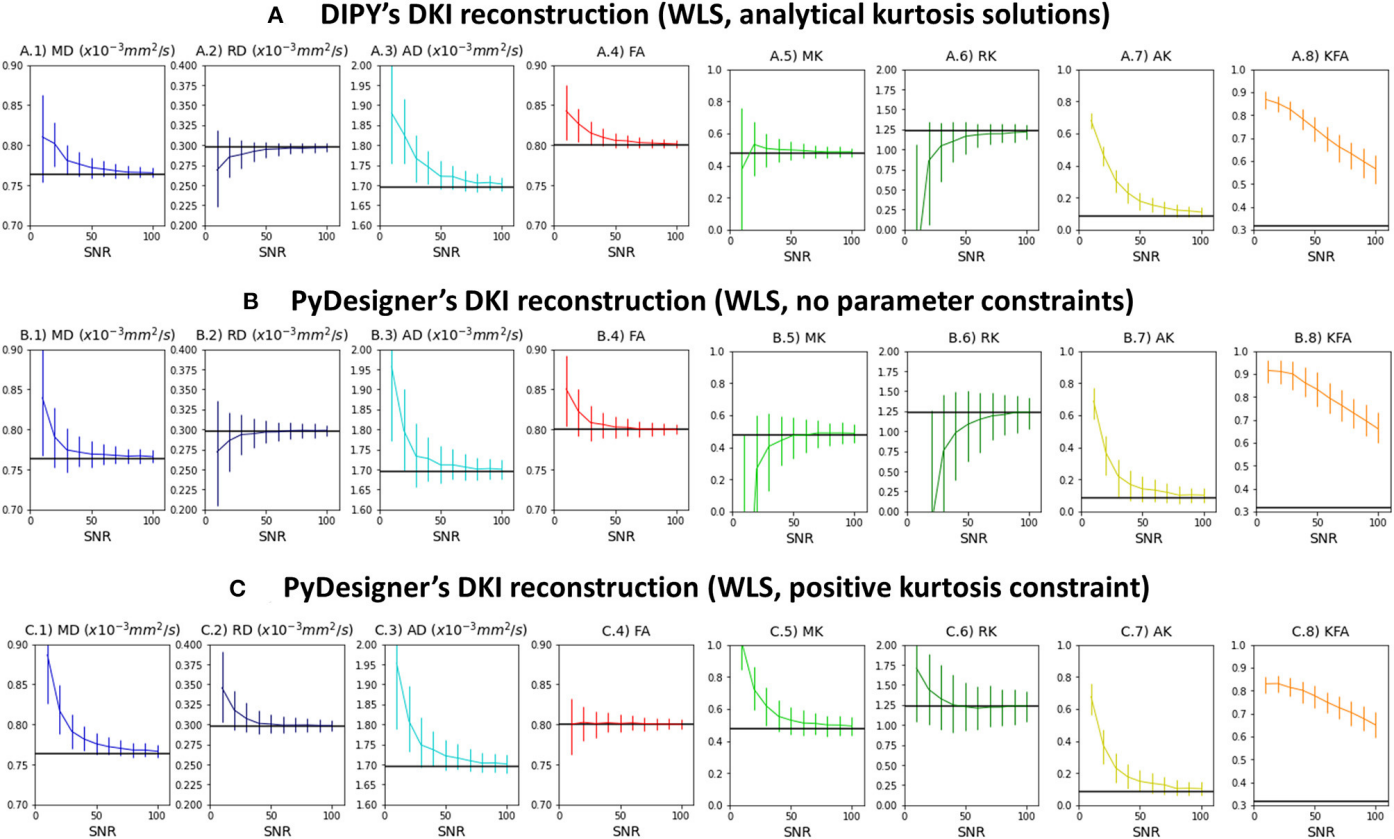
Median and interquartile ranges of the standard kurtosis parameter estimates computed from the synthetic signals of voxel case 2 corrupted with Rician noise—values are plotted as a function of signal SNR. **(A)** Estimates obtained by DIPY's DKI NLS fit. **(B)** Estimates obtained by PyDesigner WLS fit with no parameter constraints. **(C)** Estimates obtained by PyDesigner WLS fit with directional kurtosis values constraint to positive values. From left to right panels, Figures shows the results for the mean diffusivity (A.1,B.1,C.1), radial diffusivity (A.2,B.2,C.2), axial diffusivity (A.3,B.3,C.3), fractional anisotropy (A.4, B.4, C.4), mean kurtosis (A.5,B.5,C.5), radial kurtosis (A.6,B.6,C.6), axial kurtosis (A.7,B.7,C.7), and kurtosis fractional anisotropy (A.8,B.8,C.8). On each panel, the noise free estimates is marked by the black line.

Figure 3 shows the median and interquartile ranges of DKI estimates from synthetic signals of voxel case 2 corrupted with Rician noise as a function of simulations nominal SNR (results for other voxel cases can be easily reproduced from the provided jupyter notebooks). For all estimates, interquartile ranges and deviations between the median and noise free estimates (marked by the black line) are higher from lower SNRs. The SNR dependency of these statistical parameters are similar between DIPY's DKI and PyDesigner estimators with no parameter constraints (Figures 3A,B). MK, RK, and AK is underestimated by noise when these two estimators are used. When apparent kurtosis values are forced to positive values (Figure 3C), MK, RK, and AK estimates are overestimated for lower SNRs; however, the magnitude deviations between median and noise free values are similar across all tested DKI estimators. For all estimators, KFA estimates show to be highly biased by Rician noise even for a high SNR of 100.

### 3.2. Example DKI Contrasts

**Figure 4:**
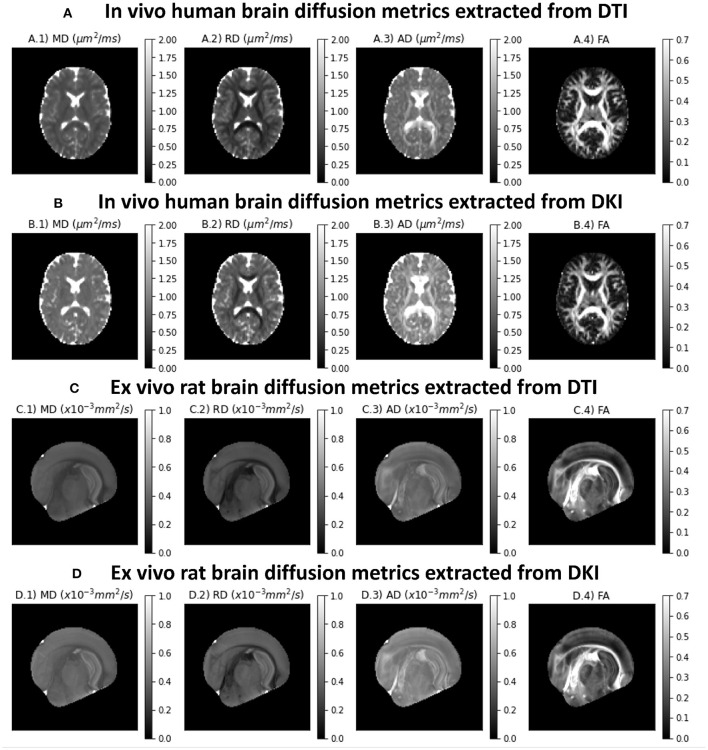
Standard diffusion metrics for a representative axial slice of the the two CFIN datasets and extracted from both DTI and DKI models implemented in DIPY: **(A)** DTI diffusion metrics for the *in vivo* human brain dataset; **(B)** DKI diffusion metrics for the *in vivo* human brain dataset; **(C)** DTI diffusion metrics for the *ex vivo* rat brain dataset; **(D)** DKI diffusion metrics for the *ex vivo* rat brain dataset;. Left to right panels show the maps of mean diffusivity (A.1,B.1,C.1,D.1), radial diffusivity (A.2,B.2,C.2 D.2), axial diffusivity (A.3,B.3,C.3,D.3), and Fractional Anisotropy (A.4,B.4,C.4,D.4).

Diffusion tensor metrics extracted from an axial slice of the *in vivo* human CFIN datasets are shown in the upper panels of Figure 4A. Upper panels show the diffusion metrics extracted from the DTI model, while Figure 4B shows the diffusion metrics extracted from the DKI model. Figures 4C,D shows the diffusion tensor metrics from the *ex vivo* rat brain for both DTI and DKI fits, respectively. As expected, all diffuvisities of the *ex vivo* rat brain dataset are lower than the *in vivo* human brain dataset. Despite this for both specimens: MD maps show low contrast between gray and white matter; lower RD and higher AD values are present on white matter regions; diffusion fractional anisotropies show higher values in white matter, particularly for regions corresponding to aligned white matter fiber bundles. MD, AD and RD estimates from the DTI model show lower values in comparison to the measures extracted from DKI (Figures 4A,C vs. B,D).

**Figure 5:**
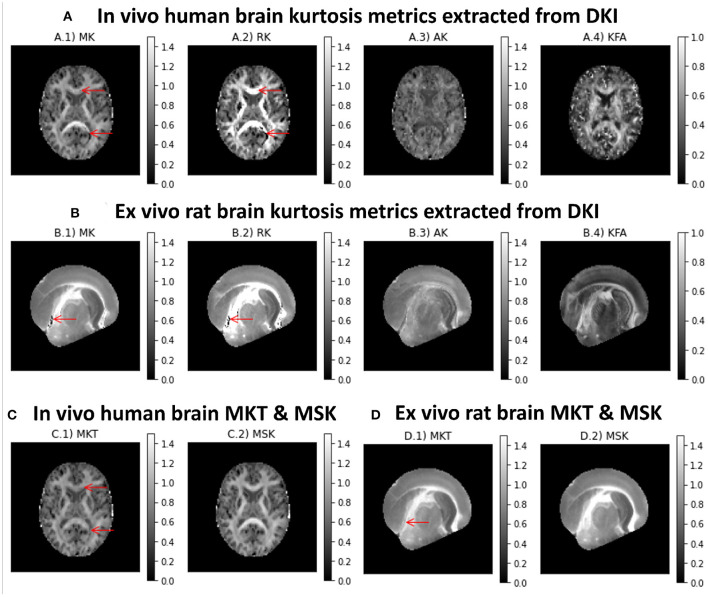
Kurtosis metrics for a representative axial slice of the two CFIN datasets computed using the DIPY's DKI and MSDKI implementations: **(A)** DKI diffusion metrics for the *in vivo* human brain dataset (A.1 Mean Kurtosis, A.2 Radial Kurtosis, A.3 Axial Kurtosis, A.4 Kurtosis Fractional Anisotropy); **(B)** DKI diffusion metrics for the *ex vivo* rat brain dataset (B.1 Mean Kurtosis, B.2 Radial Kurtosis, B.3 Axial Kurtosis, B.4 Kurtosis Fractional anisotropy); **(C)** mean kurtosis tensor and mean signal kurtosis maps for the *in vivo* human brain dataset (C.1, C.2, respectively); **(D)** mean kurtosis tensor and mean signal kurtosis maps for the *ex vivo* rat brain dataset (D.1,D.2, respectively).

Different kurtosis-based metrics are shown in Figures 5A,B for both *in vivo* human and *ex vivo* rat brains datasets, respectively. MK presents higher intensities in white matter (Figures 5A.1,B.1. On the other hand, RK shows values higher than the AK (Figures 5A.2/B.2 vs. A.3/B.3. Although diffusion-weighted data was pre-processed using the PCA denosing and Gibbs unringing algorithms, MK and RK maps still present implausible low kurtosis values in deep white matter (e.g., voxels pointed by red arrows in Figure 5). Kurtosis fractional anisotropy (KFA, Figures 5A.4,B.4) shows different contrast than the standard FA map shown in Figures 4A.4,B4,C4,D4. Mean kurtosis tensor (MKT) estimates show similar contrast to the MK map; however, MKT white matter estimates seem to be less affected by implausible low kurtosis values (Figures 5C.1,D.1). Mean signal kurtosis maps (Figures 5C.2,D.2) are also similar to MK and MKT maps; however, white matter estimates are absent from implausible negative values.

### 3.3. Comparison With pyDesigner DKI Implementation

**Figure 6:**
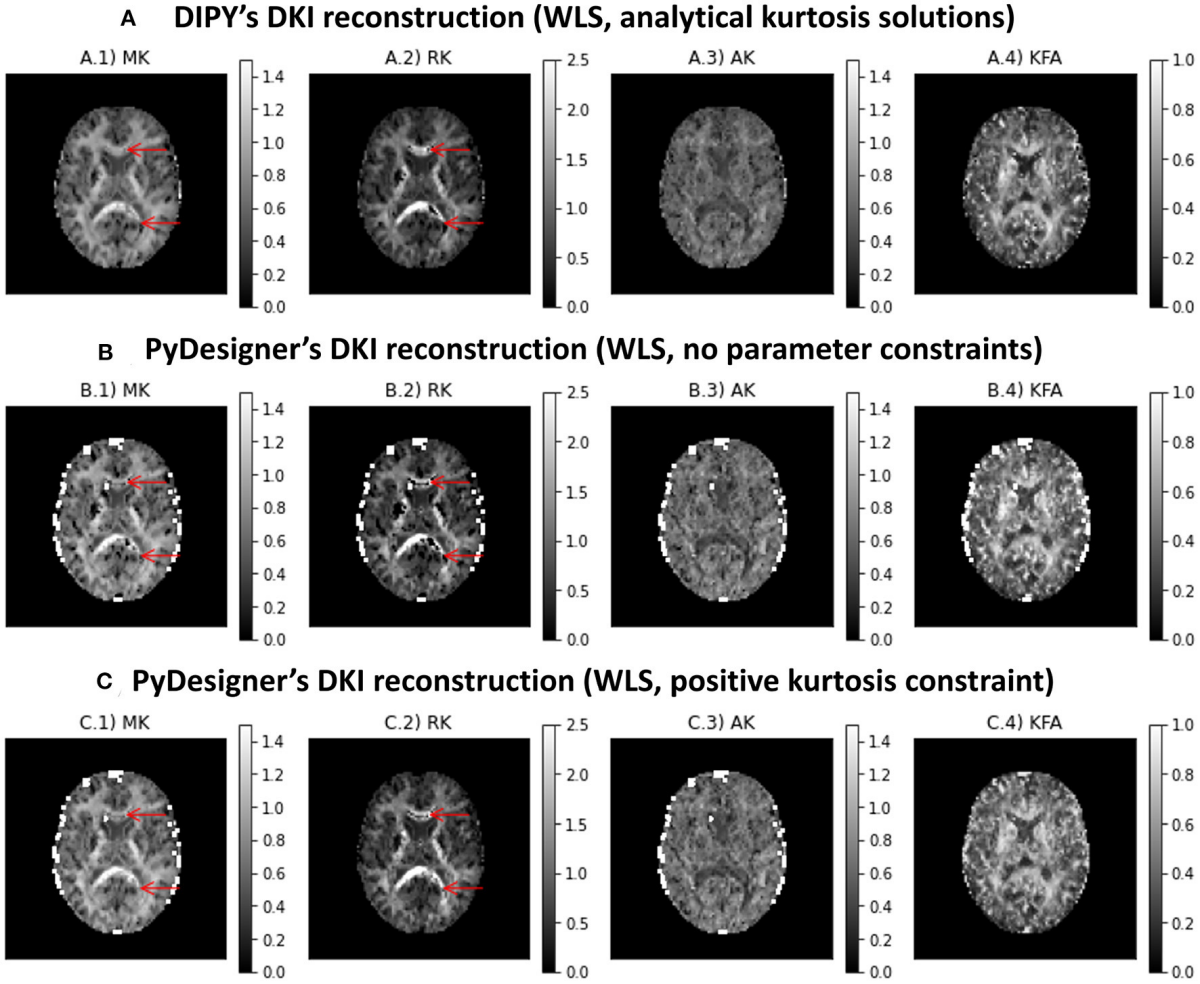
Kurtosis maps obtained from a subset of the *in vivo* human brain dataset using three different estimators: **(A)** DIPY DKI NLS estimator; **(B)** PyDesigner with no parameter constraints; **(C)** PyDesigner constraining the apparent directional kurtosis values to positive values. Left to right panels show the maps of mean kurtosis (A.1,B.1,C.1,D.1), radial diffusivity (A.2,B.2,C.2,D.2), axial diffusivity (A.3,B.3,C.3,D.3), and Fractional Anisotropy (A.4,B.4,C.4,D.4).

Figure 6 shows the kurtosis maps obtained from the subset of the *in vivo* human brain dataset using DIPY DKI implementations and PyDesigner software (Jensen et al., 2005, 2016; Jensen and Helpern, 2010; Fieremans et al., 2011; Tabesh et al., 2011; Glenn et al., 2015b; Ades-Aron et al., 2018; McKinnon et al., 2018; Moss et al., 2019; Moss and Jensen, 2021). In general, the results obtained from different estimators are in agreement (with the exception of some high kurtosis estimates from PyDesginer in regions near the parenchyma). We also find that implausible negative kurtosis in white matter can be suppressed when constraining kurtosis to positive values (see red arrows in Figure 6C.2); however, corrected values show to present lower values in comparison to adjacent white matter voxels.

### 3.4. Example DKI-Based Microstructural Model Contrast

**Figure 7:**
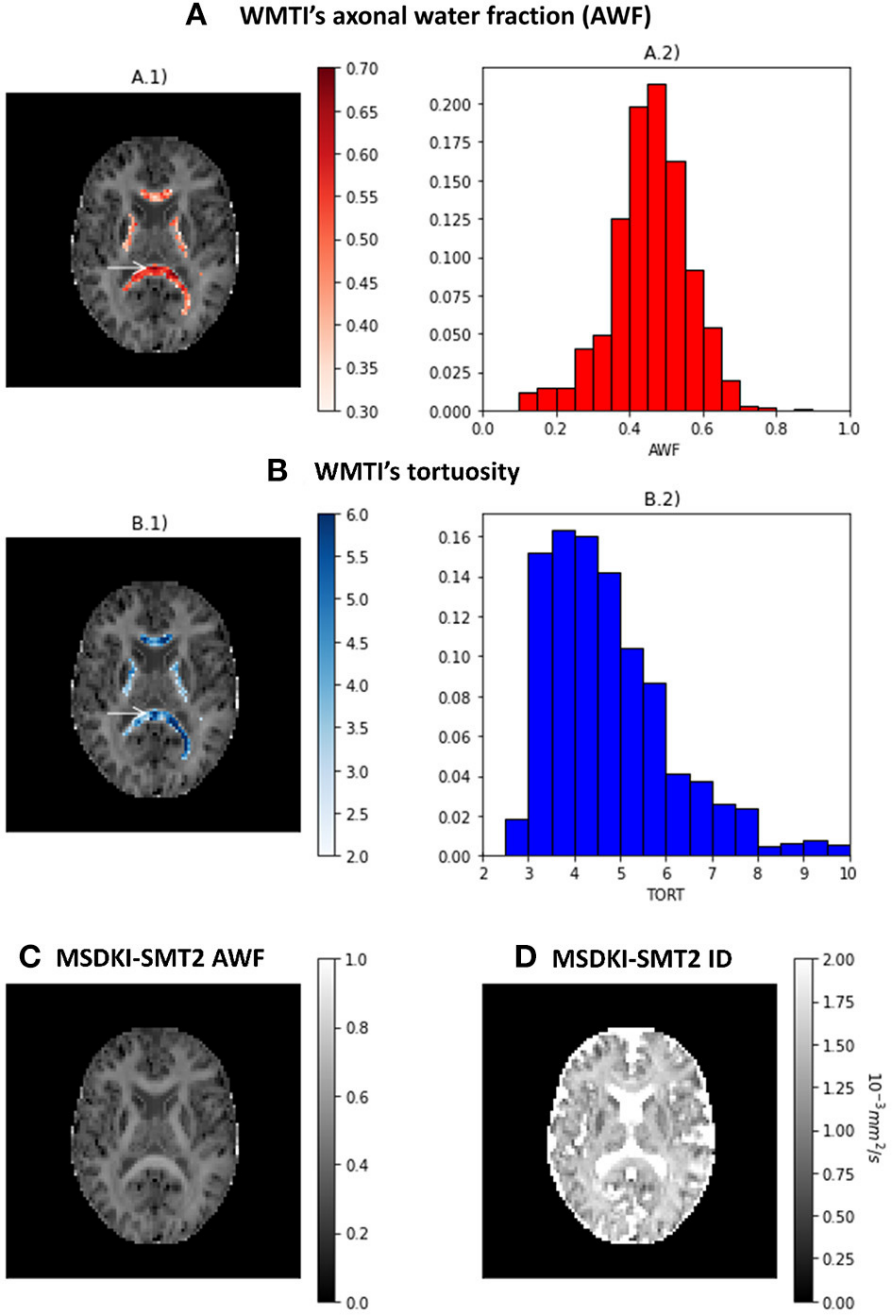
Metrics from the kurtosis-based microstructural models: **(A)** Axonal water fraction (AWF) estimates from the white matter tract integrity model—(A.1) shows the AWF estimates of well-aligned fiber regions overlaid on a top of the mean signal kurtosis image, while (A.2) shows the histograms of AWF estimates for well-aligned fiber regions. **(B)** Tortuosity (TORT) estimates from the white matter tract integrity model—(B.1) shows the TORT estimates of well-aligned fiber regions overlaid on a top of the mean signal kurtosis image, while (B.2) shows the histograms of TORT estimates for well-aligned fiber regions. **(C)** Axonal water fraction (AWF) estimates from the spherical mean technique converted from the MSDKI model. **(D)** Intrinsic diffusivity (ID) estimates from the two-compartmental spherical mean technique converted from the MSDKI model.

The results of the two kurtosis-based microstructural models are presented in Figure 7. Axonal water fraction (AWF) and tortuosity estimates from the WMTI model are plotted on well-aligned white matter regions in panels A and B, together with their histograms that reveals similar value ranges to those reported on the original WMTI paper (Fieremans et al., 2011). Figures 7C,D show the AWF and intrinsic diffusivity estimates obtained by converting the MSDKI parameters to the SMT2 (MSDKI-SMT2) model parameters (Equations 22, 21). AWF map from the MSDKI-SMT2 model presents a similar contrast than the MSK map (Figure 5C.2).

### 3.5. Evaluating and Comparing Goodness of Fit and Reliability of DTI and DKI

**Figure 8:**
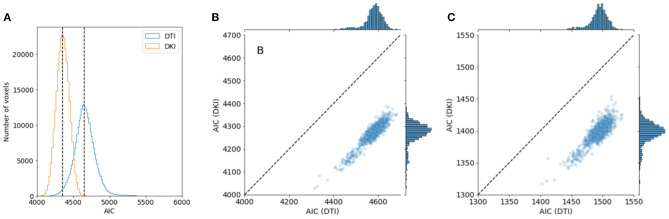
Comparing DTI And DKI goodness of fit in the Human Connectome Project dataset. Within a typical single individual **(A)**, the distribution of AICs displayed. Dashed line indicates the median of each distribution. **(B)** AIC is also consistently lower for DKI than for DTI for all subjects in the dataset who have measurements of DWI. **(C)** This holds, though the effect is substantially smaller, when the DTI model is fit only to data with *b* = 1, 000 *s*/*mm*^2^.

We compared the performance of the DKI and DTI model in a large sample: the 1,064 individuals from the HCP 1,200-subject release that had completed the entire dMRI measurement. As a measure of model goodness of fit, we computed Akaike's information criterion (AIC). We found that the median AIC is lower for DKI than for DTI in all subjects, indicating that DKI has a better goodness of fit (Figure 8). This was true both when the DTI model was fit to all of the b-values in the data, but also when DTI was fit only to the high SNR b-value tier of *b* ≈ 1, 000*s*/*mm*^2^.

**Figure 9:**
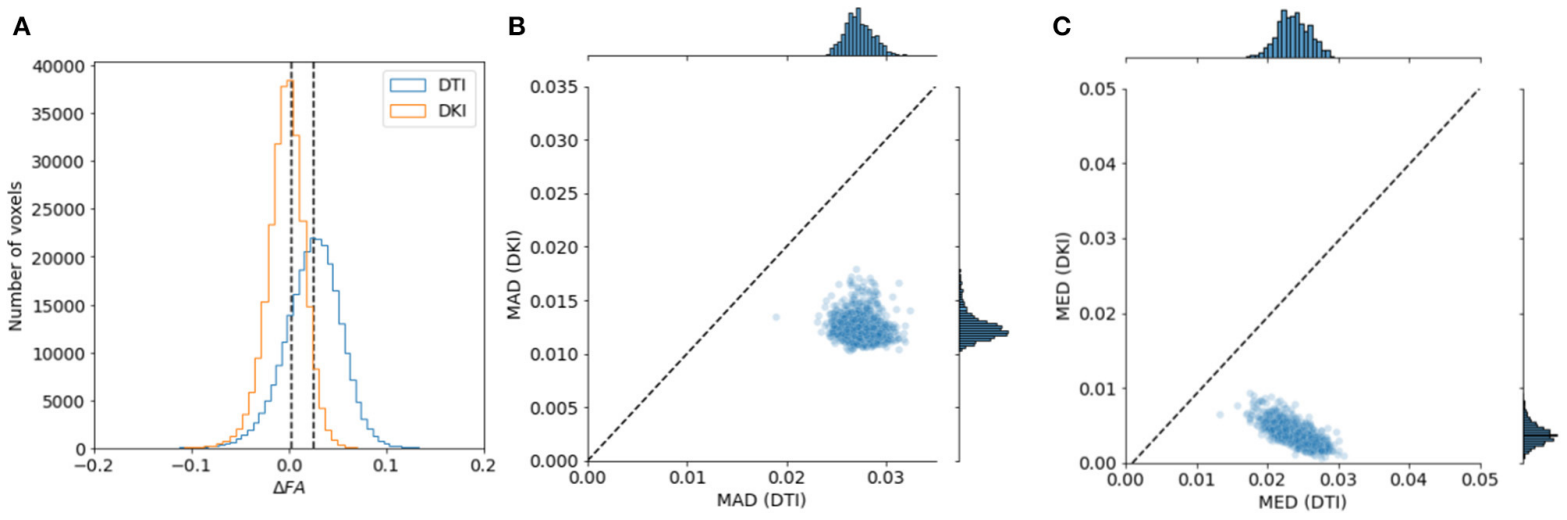
Comparing DTI And DKI FA variability in the Human Connectome Project dataset. We compared FA for DTI and DKI in different subsets of the data , divided by b-value. In each voxel in the white matter, we computed the difference in FA (Δ FA) between the subsets and quantified the properties of the distribution of Δ FA. **(A)** For example, within a representative subject, we find that the median absolute difference (MAD) is smaller for DKI than for DTI (the distribution of Δ FA is narrower). We also find that the median Δ FA is closer to 0. This is consistent in all of the subjects in the sample, both for MAD **(B)** and for median **(C)**.

**Figure 10:**
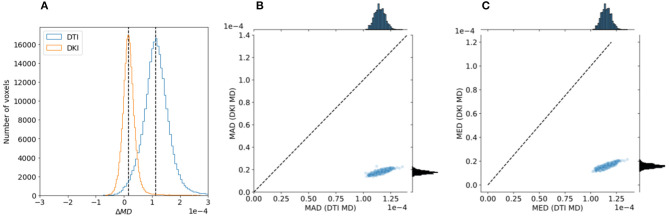
Comparing DTI And DKI MD variability in the Human Connectome Project dataset. A similar analysis to Figure 9 was conducted with MD, comparing two partially overlapping subsets of data to each other. Here as well, we find that within an individual example subject **(A)**, as well as in the entire sample, both the mean absolute deviation **(B)** and median value **(C)** are higher for DTI than for DKI.

To evaluate the reliability of the two models, we compared the stability in their estimates of the derived FA value and MD. As explained in section 2.1.4, this measure can be computed with both of the models, and their interpretation would be similar, even when values derived from the different models may differ. We used a strategy previously used in comparing these indices derived from the two models in data collected in rat brain (Veraart et al., 2011): data is sub-sampled to different b-values. For DTI, we assessed FA/MD in *b* ≈ 1, 000*s*/*mm*^2^ and in a combination of *b* ≈ 1, 000+2, 000*s*/*mm*^2^. In DKI, we assessed FA/MD in combinations of *b* ≈ 1, 000+2, 000*s*/*mm*^2^ and *b* ≈ 1, 000+3, 000*s*/*mm*^2^. We find that FA/MD reliability is consistently higher for DKI than for DTI (Figures 9, 10). This is true both in terms of the magnitude of the differences as well as the degree to which they tend to be centered on zero.

## 4. Discussion

Diffusional kurtosis Imaging (DKI) is a straightforward expansion of standard diffusion tensor imaging (DTI).

Here, we provide a well-tested reference implementation of DKI model fitting and related techniques in the DIPY project (Garyfallidis et al., 2014) (100% test coverage; Zhu et al., 1997). The implementation is feature complete: it includes several different methods for fitting the basic DKI model and compute derived quantities, as well as relevant extensions to the model: the mean signal DKI (MSDKI) model (Henriques, 2018), WMTI (Fieremans et al., 2011), and the MSDKI-SMT models (Henriques et al., 2019). A reference implementation of these methods that is comprehensive, thoroughly-tested and well-documented is an important accelerant for subsequent scientific research. It provides a proving ground for new methods and a basis for comparison between methods. The fact that DIPY is managed as an open software library, where issues can be publicly reported, discussed and addressed, means that errors can be surfaced by any user of the software. The open-source code means that these issues can be demonstrated and fixed directly by reference to the code itself. This is important for correctness of the implementation, and it also promotes the reproducibility of results obtained using this implementation (Rokem et al., 2018). The modularity and object-oriented design of the software means that statistical procedures that are implemented in one model can be readily translated to other models. For example, we demonstrate here the use of cross-validation for model evaluation (Rokem et al., 2015). The DIPY application programming interface provides uniform methods for resampling such that the model is fit in some directions and predicted in other directions. This software architecture is extensible. We have already used this fact to extend DKI. But it also means that others can rely on the architecture to build future developments.

### 4.1. Findings

To demonstrate the utility of our software, we analyzed several different datasets. Numerical simulations were first used to demonstrate the sensitivity of the DKI method and the software in known microstructural configurations. Particularly, based on these simulations, we demonstrate that DKI does not only provides a quantification of non-Gaussian diffusion but also decouples non-Gaussian diffusion effects from standard diffusion tensor metrics–a reason why DKI diffusion tensor estimates more closely match their ground truth estimates than the DTI tensor estimates (Figure 1). Simulations were also used to illustrate that, while systems comprising different components with distinct diffusivities and configurations can present very similar diffusivities, kurtosis estimates can help distinguish them by providing information on diffusion heterogeneity (Figure 2). Our simulations also reproduce the kurtosis geometries exploration of Henriques et al. (2015) which revealed that maximum kurtosis values are present perpendicular individual fibers even in crossing configurations, and confirmed that MSK estimates are invariant to the directional configuration of tissue compartments. We also confirmed that kurtosis fractional anisotropy provides different information than the standard diffusion fractional anisotropy (Glenn et al., 2015b; Hansen and Jespersen, 2016b; Hansen, 2019); however, we found that KFA can be highly corrupted by noise biased by noise even at high SNRs (Figure 3).

We used the CFIN human brain dataset to provide examples of the contrasts provided by DKI (Hansen and Jespersen, 2016a). This is a dataset that is directly and openly accessible to anyone through the DIPY dataset interface, so the figures using this data can be reproduced with code that we provide (and is also provided as part of the DIPY documentation, both for standard DKI^2^ and for MSDKI ^3^). This data is used in tandem with supporting methods that address some of the limitations of the method (see below): to produce the maps shown in Figures 4, 5, we used both denoising (Veraart et al., 2016b) and Gibbs ringing removal (Kellner et al., 2016; Henriques, 2018), both implemented in DIPY. Based on this sample dataset, we demonstrate the typical contrasts of Mean, axial and radial diffusivities and kurtosis. We illustrate that MK, MKT, and MSK present similar contrasts (consistent to what was reported by Hansen et al., 2013), but MSK was shown to be more robust to image noise and artifacts. We also ran the DIPY DKI implementations on a open access *ex vivo* rat brain dataset (Hansen and Jespersen, 2016a), confirming that the implementations produce stable results from data acquired on both clinical and pre-clinical scanner and on different species (*in vivo* and *ex vivo*).

The CFIN human brain dataset were also used to illustrate the estimates obtained from the implemented kurtosis-based microstructural models (Figure 7). Axonal volume fraction and extracellular tortuosity estimates from the white matter tract integrity model (WMTI) showed similar value ranges than reported on the original WMTI paper (Fieremans et al., 2011). As one may expect from the theory (e.g., equation 22), we also highlighted that the axonal water fraction maps obtained for the MSDKI-SMT2 model provide similar contrast to MSK. Since previous studies showed that SMT2 assumptions do not properly represent biological tissues (Henriques et al., 2019), AWF should not be interpreted as accurate biophysical estimates of axonal water fraction. Instead, it could be a useful normalized version of MSK scaled in a range between 0 and 1.

Finally, we analyzed a large, openly available dataset provided by the Human Connectome Project (Sotiropoulos et al., 2013; Glasser et al., 2016). In this dataset, we found that DKI consistently fit the data more accurately than DTI. In addition, FA and MD derived from DKI showed less variability across different sub-samples of the data. Considering these two facts, we conclude that in this dataset, FA and other metrics should be computed using the DKI model. These findings are important in the context of the HCP dataset, as this data is likely to be analyzed by many other researchers. In addition, several efforts are currently underway to collect similar large-scale datasets with multiple diffusion weighting values (e.g., Alexander et al., 2017; Jernigan et al., 2018), and similar conclusions may apply in these datasets as well.

### 4.2. Related Work

There are several other software implementations of DKI that are available (see Table 2 for a comparison). These include implementations in the DKE software (Tabesh et al., 2011), available through NITRC ^4^ and ExploreDTI (Leemans et al., 2009). But neither of these software projects is open-source or provided through an OSI-approved license, limiting their broad use. In addition, they both require the proprietary Matlab software platform. While MATLAB is widely available in most academic environments in the developed world, it is less accessible in developing countries (Ramachandran, 2016) and in extra-academic uses of diffusion MRI (e.g., in industry). Other Python-based software that provides open-source and OSI-approved licensed software are DESIGNER (Ades-Aron et al., 2018) and the recently-released pyDesigner (Jensen et al., 2005, 2016; Jensen and Helpern, 2010; Fieremans et al., 2011; Tabesh et al., 2011; Glenn et al., 2015b; Ades-Aron et al., 2018; McKinnon et al., 2018; Moss et al., 2019; Moss and Jensen, 2021). A comparison of the DIPY implementation to the pyDesigner implementation provided a very close match (Figure 6). Given that the implementations were done completely independently, this agreement provides a sign of the robustness of DKI across different software (Kruper et al., 2021). Finally, the recent version of the mrtrix software (Tournier et al., 2019) also includes an implementation of DKI estimation. The software presented here adds to these software projects in that it includes Python-based implementations that are, on the one hand, completely open-source and gratis for use in any context. On the other hand, using the Python programming language enhances the readability of the code and its understanding by researchers from many different backgrounds (e.g., relative to scientific software implemented in compiled languages, such as C/C++). The software is specifically designed to enable extensions and further developments on top of the existing methods. These design considerations have allowed our development team to expand the original implementation of DKI to include many different additional methods, described above.

**Table 2:** Some freely available software libraries that implements DKI.

**Software**	**License**	**Language**	**Micro?**	**ODF?**	**References**
Designer^5^	Mozilla public license	Python (also requires MATLAB, mrtrix, and FSL).	Yes	No	Ades-Aron et al., 2018
pyDesigner^6^	Mozilla Public license	Python (Also requires FSL and mrtrix)	Yes	Yes	Jensen et al., 2005, 2016; Jensen and Helpern, 2010; Fieremans et al., 2011; Tabesh et al., 2011; Glenn et al., 2015b; Ades-Aron et al., 2018; McKinnon et al., 2018; Moss et al., 2019; Moss and Jensen, 2021
mrtrix^7^	Mozilla public license	C/C++	No	No	Tournier et al., 2019
Diffusion kurtosis estimator (DKE)^8^	Custom	MATLAB	Yes	Yes	Tabesh et al., 2011
DIPY^9^	BSD	Python	Yes	No	Garyfallidis et al., 2014

*The table specifies the license and programming language of implementation, whether the software implements DKI-based microstructure models and DKI-based orientation distribution functions (ODF) and the relevant references to cite when using the software*.

### 4.3. Limitations and Future Work

As we highlight in this work, DKI can provide more accurate diffusion estimates than DTI in addition to measures of diffusional kurtosis. However, it is important to note, fitting DKI requires data from multi-shell b-values, and thus it cannot be used to analyze data acquired using single non-zero b-value acquisitions. Moreover, since it involves the estimation of a larger number of parameters than DTI, DKI can provide less precise (i.e., noisier) estimates. Moreover, our simulations (Figure 3) shows that DKI parameters can not only suffer from low precision but also biased by Rician noise even at typically diffusion data SNRs ( 20-40). As illustrated in Figure 5, thermal noise biases can manifest as implausible negative estimates “black voxels” in standard DKI maps, particularly in regions where diffusivities are low (Tabesh et al., 2011; Henriques, 2012; Kuder et al., 2012; Veraart et al., 2013). Implausible negative kurtosis estimates can also originate from effects of different image artefact such as Gibbs Ringing artefacts as explained by Perrone et al. (2015) and Veraart et al. (2016a). To to minimize the effects of noise and artefact, in this work we decided to used state-of-the denoising and artefact suppression algorithms (Kellner et al., 2016; Veraart et al., 2016b); however, other pre-processing techniques in DIPY could be used, including the threshold-based PCA denoising (Manjón et al., 2013), the non-local means denoising filter (Coupé et al., 2011) and the self-supervised denoiser Patch2Self (Fadnavis et al., 2020). Particularly, Fadnavis et al. demonstrated that Patch2Self outperforms current implementations of low-rank method approximations, such as MP-PCA (Fadnavis et al., 2020). Implausible kurtosis estimates can also be mitigated by excluding data outliers Chang et al., 2005; Tax et al., 2015), imposing constraints (Tabesh et al., 2011; Kuder et al., 2012) (as also shown in Figure 6), and readjusting b-value=0 data points (Zhang et al., 2019). Alternatively, if only interested in isotropic kurtosis measures, one could opt to use of the MSK estimates which provide more precise quantification of non-Gaussian diffusion in low-diffusivity regions (c.f. Figure 5). In addition, MSK can be used to regularize the full DKI providing more robust kurtosis tensor derived metrics (Henriques et al., 2021). In future studies, DIPY can provide a useful framework for the comparison of all these different noise and artefact suppression techniques and for the development of novel strategies.

As with other techniques based on the diffusion-weighted signal cumulant expansion, DKI estimates may be biased by high-order-term effects not considered by the expansion truncation (Chuhutin et al., 2017). While higher-order-effect in diffusion tensor metrics are minimized by DKI (c.f. Figure 1B), these can introduce the deviations between kurtosis tensor estimates and their ground truth values. Despite these accuracy issues, fitted kurtosis tensors still present a fair description of the 3D information of the kurtosis tensor as shown in Figure 1C. Although DKI may provide robust information from microstructural model fitting (Fieremans et al., 2011; Jespersen, 2018; Jespersen et al., 2018), it is important to note that the higher-order-term biases on DKI can propagate to DKI-based microstructural estimates. Therefore, in future studies it will be of interest to combine our implemented strategies to the full analytical derivations of different microstructural models. For instance, the more robust DKI-based estimates could be used as the initial guess estimates for the more complex non-linear fitting procedures required by some current microstructural models. Moreover, we expect that our DKI microstructural models could be expanded to remove some of the model constraints assumed by WMTI and SMT2 models (e.g., incorporating tissue dispersion and removing the lower intra-cellular diffusivity constraint of the WMTI model (Jelescu et al., 2016; Jespersen, 2018; Jespersen et al., 2018; Novikov et al., 2018b).

While DKI can provide additional information to DTI, the non-Gaussian diffusion information provided by single diffusion encoding (SDE) multi-shell acquisitions can originate from multiple sources, and thus limiting kurtosis specificity (Szczepankiewicz et al., 2016; Henriques et al., 2020a). For example, kurtosis decreases in gradual degeneration processes (e.g., healthy ageing, Alzheimer's and Parkison's diseases, multiple sclerosis) are typically attributed to a reduction in individual fibers' diffusion anisotropy (Falangola et al., 2008; Wang et al., 2011; Struyfs et al., 2015; Andersen et al., 2020). On the other hand, kurtosis increases in more abrupt damaged microstructural tissues (e.g., ischemia, traumatic brain injury) can occur due to cytogenic and vasogenic effects (Hui et al., 2012; Zhuo et al., 2012). To increase the specificity of kurtosis, several studies have used advanced diffusion encoding sequences to decouple different kurtosis sources (e.g., Szczepankiewicz et al., 2016, 2020; Westin et al., 2016; Topgaard, 2017; Henriques et al., 2020a,b). Due to the high potential of these techniques, procedures for reconstructing diffusion-weighted data acquired from advanced diffusion sequences are currently being incorporated in DIPY.

The present work focused on the use kurtosis to probe microstructural and biophysical properties of the tissue in different locations within the white matter. However, DKI can also provide information to identify the trajectories of white matter bundles through the brain, connecting local or remote parts of the brain to each other through computational tractography (Lazar et al., 2008; Jensen et al., 2014; Glenn et al., 2015a, 2016; Henriques et al., 2015). To implement the DKI-based tractography in DIPY we would require an implementation of an analytical solution that relates the diffusion and kurtosis tensors to the tissue orientation distribution function (ODF). This will be included in future releases of DIPY. For the time being, DIPY provides many other methods to compute ODFs, including from DTI, as well as from constrained spherical deconvolution (Tournier et al., 2007) (including a multi-shell variant of this method; Jeurissen et al., 2014).

## Data Availability Statement

Publicly available datasets were analyzed in this study. This data can be found here: The CFIN dataset is available through https://datadryad.org/stash/dataset/10.5061/dryad.9bc43 and can also automatically be downloaded using DIPY's data fetcher module. The Human Connectome Project dataset can be accessed via https://www.humanconnectome.org/.

## Author Contributions

RH led the implementation of the software. MC, MM, SK, EG, and AR contributed to, and reviewed the software implementation. JY and EH tested the software in real datasets and contributed to the implementation of microstructural modeling API. JK conducted analysis HCP data. RH and AR conducted simulations, analyzed data, and wrote the manuscript, with feedback from all other authors.

## Conflict of Interest

The authors declare that the research was conducted in the absence of any commercial or financial relationships that could be construed as a potential conflict of interest.

## Footnotes

^1^http://gsoc2015dipydki.blogspot.com/2015/07/rnh-post-8-computing-perpendicular.html

^2^https://dipy.org/documentation/latest/examples_built/reconst_dki/

^3^https://dipy.org/documentation/latest/examples_built/reconst_msdki

^4^https://www.nitrc.org/projects/dke/

^5^https://github.com/NYU-DiffusionMRI/DESIGNER

^6^http://pydesigner.readthedocs.io/

^7^https://www.mrtrix.org/

^8^https://www.nitrc.org/projects/dke

^9^https://dipy.org/
